# Multidimensional efficacy characterization of xeligekimab in moderate-to-severe plaque psoriasis: regional responses, treatment targets, and patient-reported outcomes from a randomized controlled trial

**DOI:** 10.3389/fimmu.2026.1917350

**Published:** 2026-09-18

**Authors:** Kun Huang, Yi Zhao, Chunjun Yang, Jianzhong Zhang

**Affiliations:** 1 Dermatology Department, The First Affiliated Hospital of Chongqing Medical University, Chongqing, China; 2 Dermatology Department, Beijing Tsinghua Changgung Hospital, Beijing, China; 3 Department of Dermatology and Venereology, The Second Affiliated Hospital of Anhui Medical University, Hefei, Anhui, China; 4 Dermatology Department, Peking University People’s Hospital, Beijing, China

**Keywords:** patient-reported outcomes, plaque psoriasis, *post hoc* analysis, treatment targets, xeligekimab

## Abstract

**Background:**

Xeligekimab, a monoclonal antibody targeting interleukin-17A (*IL-17A*), has demonstrated high efficacy in treating moderate-to-severe plaque psoriasis. However, the detailed response patterns across anatomical regions, treatment targets, and patient-reported outcomes remain incompletely elucidated.

**Methods:**

This *post hoc* analysis of a phase 3, randomized, double-blind, placebo-controlled trial (CHICTR2100043223) included 420 Chinese patients randomized 2:1 to receive subcutaneous xeligekimab 200 mg every 2 weeks or placebo for 12 weeks, followed by open-label xeligekimab through week 52. Regional Psoriasis Area and Severity Index (PASI) responses across four anatomical sites, treatment targets [ideal target: PASI 90 or absolute PASI ≤3 or body surface area (BSA) ≤1% or Physician’s Global Assessment (PGA) 0/1; acceptable target: PASI 75 or absolute PASI ≤5 or BSA ≤3% or PGA 0/1/2; sustained remission: BSA = 0% or PGA = 0 for ≥6 months], and Dermatology Life Quality Index (DLQI) domain-specific improvements were comprehensively analyzed.

**Results:**

Xeligekimab produced rapid improvements across all PASI dimensions by week 4, with induration (−60.1%), scaling (−61.9%), and erythema (−50.3%) versus −5.3% to −7.7% for placebo (all *P* < 0.001). By week 12, reductions reached −91.1%, −90.9%, and −82.3%, respectively. Regional analysis showed that head lesions responded most rapidly (PASI 90: 84.2% at week 12), with week 52 regional PASI 90 rates ranging from 81.2% to 92.1%. Ideal and acceptable target achievement rates reached 86.6% and 94.9% at week 12 versus 4.6% and 9.9% for placebo (both *P* < 0.001), increasing to 91.0% and 98.0% by week 52. The median time to sustained remission was 48 weeks for xeligekimab. By weeks 52 and 60, sustained remission rates reached 50.1% and 54.1%, respectively. Patient-reported outcomes demonstrated rapid and comprehensive improvement across all DLQI functional dimensions by week 4, with low-impact rates exceeding 90% by week 12 for physical symptoms (97.0% for symptoms, 92.0% for pruritus), psychological burden (98.0%), daily activities (97.1%–98.2%), social functioning (98.0%), work/study (97.0%), and treatment burden (99.0%), all significantly superior to placebo (all *P* < 0.001).

**Conclusion:**

Xeligekimab provides rapid symptom resolution across all clinical dimensions, high and sustained achievement of treatment targets, and comprehensive restoration of quality of life.

**Clinical Trial Registration:**

https://www.chictr.org.cn, identifier CHICTR2100043223.

## 1 Introduction

Plaque psoriasis is a chronic, immune-mediated inflammatory skin disease affecting approximately 2%–3% of the global population (1). Beyond visible skin lesions, psoriasis imposes substantial psychological, social, and occupational burdens. Depression and anxiety affect 20%–21% of patients, while impairments in daily activities and work productivity are documented in the literature (2, 3). Importantly, traditional clinical endpoints such as the Psoriasis Area and Severity Index (PASI) and body surface area (BSA) do not always reflect the patient-perceived disease burden. Studies have shown disconnect between objective severity measures and subjective quality-of-life impairment (4, 5). This discrepancy underscores the need for comprehensive, multidimensional patient-reported outcome (PRO) assessments, particularly in the era of highly effective biologic therapies in which treatment goals extend beyond skin clearance.

The IL-23/Th17 axis plays a central role in psoriasis pathogenesis, with *IL-17A* driving keratinocyte proliferation and chronic inflammation (6). Anti-*IL-17A* monoclonal antibodies, including ixekizumab, secukinumab, and bimekizumab, have demonstrated rapid onset and high PASI 90 response rates exceeding 70%–80% by weeks 12–16 in pivotal trials (7). Network meta-analyses rank *IL-17* inhibitors among the fastest-acting biologics, with sustained efficacy over long-term treatment (8). However, three knowledge gaps persist. First, regional response variability across anatomical sites has not been systematically quantified, despite evidence that lower extremities exhibit higher treatment resistance. Second, contemporary treat-to-target strategies advocate treatment targets that encompass ideal targets (near-complete clearance), acceptable targets (substantial improvement), and sustained remission during treatment; yet, temporal trajectories and cumulative achievement rates for these stratified endpoints remain poorly characterized. Third, domain-specific improvements across the six functional dimensions of the Dermatology Life Quality Index (DLQI) have not been comprehensively evaluated for newer anti-*IL-17A* agents.

Xeligekimab is a novel anti-*IL-17A* monoclonal antibody that demonstrated high overall efficacy in a phase 3 trial in Chinese patients with moderate-to-severe plaque psoriasis (9). This *post hoc* analysis aims to characterize its multidimensional efficacy across three domains: regional PASI response kinetics by anatomical site, treatment target achievement over time, and DLQI domain-specific improvement patterns. These analyses are intended to support precision treatment strategies and patient-centered therapeutic optimization.

## 2 Methods

### 2.1 Study design and patient population

This *post hoc* analysis utilized data from a phase 3, randomized, double-blind, placebo-controlled, multicenter trial (CHICTR2100043223) that evaluated the efficacy and safety of xeligekimab in Chinese patients with moderate-to-severe plaque psoriasis. The original trial enrolled 420 patients across multiple centers in China. Eligible patients were aged 18–70 years with chronic plaque psoriasis for ≥6 months, a baseline PASI score ≥12, a Physician’s Global Assessment (PGA) score ≥3 (PGA is a static 6-point scale: 0 = clear, 1 = minimal, 2 = mild, 3 = moderate, 4 = marked, 5 = severe), BSA involvement ≥10%, and an inadequate response to conventional therapies (topical treatments, phototherapy, or systemic therapy). Patients were randomized 2:1 using an interactive web response system to receive subcutaneous xeligekimab 200 mg every 2 weeks (Q2W, *n* = 281) or matching placebo (*n* = 139) during the 12-week double-blind induction period. Subsequently, all patients entered an open-label extension phase, receiving xeligekimab 200 mg every 4 weeks (Q4W) from weeks 12 to 52, followed by an 8-week safety follow-up through week 60.

This study was conducted in accordance with the Declaration of Helsinki. Overall ethical approval was obtained from the Ethics Committee of Peking University People’s Hospital as the lead committee (approval no. 2020PHA072-001, 27 January 2021). Each of the 35 participating centers independently obtained institutional ethics approval from their respective ethics committees prior to enrolling the first participant. The studies were conducted in accordance with local legislation and institutional requirements. All participants provided written informed consent before study entry. For descriptive comparisons by body weight, patients were categorized as <75 kg or ≥75 kg. This threshold has been applied in prior real-world analyses of biologics in Chinese patients with psoriasis (10).

### 2.2 Efficacy outcome definitions

#### 2.2.1 Regional response analysis

PASI component scores (erythema, induration, and scaling) were evaluated as the mean percentage change from baseline at each visit to characterize the temporal pattern of improvement across disease dimensions. Additionally, PASI component scores for four anatomical regions (head, upper limbs, trunk, and lower limbs) were analyzed separately to assess region-specific PASI 90/100 response rates at each time point.

#### 2.2.2 Treatment target definitions

Treatment targets were defined according to the Chinese Expert Consensus on Treat-to-Target Strategy with Biologics for Psoriasis (11). This consensus stratified treatment goals into two hierarchical levels to guide clinical decision-making: ideal target and acceptable target.

Ideal target was defined as achieving any one of the following criteria: (1) PASI 90 response, (2) absolute PASI score ≤3, (3) BSA ≤1%, or (4) PGA 0/1.

Acceptable target required meeting any one of the following: (1) PASI 75 response, (2) absolute PASI score ≤5, (3) BSA ≤3%, or (4) PGA 0/1/2.

#### 2.2.3 Sustained remission definition

Sustained remission was originally defined by the National Psoriasis Foundation (NPF) as achieving an Investigator’s Global Assessment (IGA) score of 0 or BSA of 0%, maintained continuously for at least 6 months (12). Given that the endpoint of this phase 3 trial (9) was based on PGA rather than IGA, and considering that both scales represent static global assessments of overall disease severity and are regarded as interchangeable in defining complete skin clearance, we substituted PGA for IGA in our operational criteria.

The 6-month duration aligns with the NPF guideline recommendation that maintenance-phase treatment goals be reassessed at 6-month intervals (12). In the context of this trial’s Q4W dosing regimen, 6 months corresponds operationally to six 4-week treatment cycles (24 weeks). Accordingly, integrating the study design, we refined the operational definition of sustained remission as maintaining BSA = 0% or PGA = 0 continuously for ≥24 weeks. This adaptation minimally affects the assessment of sustained remission while ensuring alignment with both the NPF framework and the trial’s dosing schedule.

#### 2.2.4 Patient-reported outcomes

DLQI assessments were analyzed across six functional dimensions: physical symptoms [question 1 (Q1)], psychological well-being (Q2), daily activities (Q3, Q4, Q6, and Q9), social functioning (Q5 and Q8), work/study interference (Q7), and treatment burden (Q10). Low impact was defined as a DLQI item score ≤1 (response of “Not at all” or “A little”) for each functional dimension.

Pruritus severity was assessed using an 11-point Numerical Rating Scale (NRS), with scores of 0–3 classified as none or mild pruritus. The proportion of patients achieving low impact across each dimension was compared between treatment groups at each visit.

### 2.3 Statistical analysis

Baseline demographics and disease characteristics were summarized using descriptive statistics (mean ± SD for continuous variables; frequency and percentage for categorical variables). Regional and component-level PASI analyses, including baseline summaries, were restricted to patients with a baseline score >0 for the corresponding dimension or anatomical region. Time-to-event analyses for treatment target achievement were performed using Kaplan–Meier survival curves. For between-group comparisons of response rates (PASI response, treatment targets, and PRO low-impact proportions), chi-square tests or Fisher’s exact tests were used as appropriate, with results presented as percentages with 95% confidence intervals (CI). Data after week 12 were described descriptively. All statistical analyses were conducted using R software version 4.2.0 (Vienna, Austria). Analyses were designated as exploratory (regional PASI responses, treatment target achievement, and DLQI domain-specific outcomes). The Benjamini–Hochberg procedure was applied within four predefined, internally consistent comparison families, and Benjamini–Hochberg-adjusted *P*-values were reported together with nominal *P*-values. Two-sided adjusted *P*-values <0.05 were considered statistically significant.

## 3 Results

### 3.1 Baseline demographics and disease characteristics

A total of 420 patients were randomized (xeligekimab, *n* = 281; placebo, *n* = 139). Baseline demographics and disease characteristics were well balanced between groups (**Table 1**), with a mean age of 40.8 years, 75.2% male patients, a mean PASI score of 22.20, a mean BSA of 33.7%, and 87.1% biologic-naive. Disease severity was substantial, with 71.0% having a PGA score of 3. Regional PASI scores showed lower limbs as the most affected site (10.72), followed by the trunk (6.18), upper limbs (3.74), and head (1.72). PASI component scores were comparable across groups, with mean erythema, induration, and scaling scores of 10.01, 9.70, and 9.68, respectively. The mean baseline DLQI was 13.8, and the itch NRS score was 5.6, indicating substantial quality-of-life impairment.

**Table 1:** Patient demographics and baseline clinical characteristics.

Characteristic	Xeligekimab (*N* = 281)	Placebo (*N* = 139)	Total (*N* = 420)^a^
Age (years), mean ± SD	40.4 ± 11.79	41.4 ± 11.65	40.8 ± 11.74
Body weight (kg), mean ± SD	73.46 ± 13.565	72.43 ± 13.253	73.12 ± 13.455
Weight category, *n* (%)			
<75 kg	158 (56.2)	78 (56.1)	236 (56.2)
≥75 kg	123 (43.8)	61 (43.9)	184 (43.8)
Sex, *n* (%)			
Male	214 (76.2)	102 (73.4)	316 (75.2)
Female	67 (23.8)	37 (26.6)	104 (24.8)
Psoriasis duration (years), mean ± SD	13.17 ± 8.493	13.94 ± 9.472	13.42 ± 8.825
PASI score, mean ± SD	22.23 ± 9.066	22.12 ± 9.405	22.20 ± 9.168
BSA (%), mean ± SD	33.752 ± 18.358	33.561 ± 18.900	33.689 ± 18.517
PGA score, *n* (%)			
3	199 (70.8)	99 (71.2)	298 (71.0)
4	82 (29.2)	40 (28.8)	122 (29.0)
DLQI score, mean ± SD	14.1 ± 7.01	13.3 ± 6.89	13.8 ± 6.98
Itch NRS score, mean ± SD	5.6 ± 2.45	5.6 ± 2.34	5.6 ± 2.41
Previous biologic therapy, *n* (%)			
Yes	37 (13.2)	17 (12.2)	54 (12.9)
No	244 (86.8)	122 (87.8)	366 (87.1)
PASI regional scores			
Head score	1.72 ± 1.00	1.73 ± 1.06	1.72 ± 1.02
Upper limb score	3.74 ± 1.98	3.76 ± 2.08	3.74 ± 2.01
Trunk score	6.17 ± 3.24	6.21 ± 3.27	6.18 ± 3.25
Lower-limb score	10.75 ± 4.99	10.66 ± 5.09	10.72 ± 5.02
PASI component scores			
Induration	9.62 ± 2.42	9.86 ± 2.53	9.70 ± 2.46
Erythema	9.98 ± 2.27	10.09 ± 2.43	10.01 ± 2.32
Scaling	9.65 ± 2.67	9.74 ± 2.71	9.68 ± 2.68

*All patients were Chinese. Data are presented as mean ± SD or *n* (%). Regional PASI scores and PASI component scores (induration, erythema, scaling) were summarized in patients with a baseline score >0. SD, standard deviation; PASI, Psoriasis Area and Severity Index; BSA, body surface area; PGA, Physician’s Global Assessment; DLQI, Dermatology Life Quality Index; NRS, Numerical Rating Scale.

### 3.2 PASI component score improvements

At week 4, the mean percentage reductions from baseline were −60.1% for induration, −61.9% for scaling, and −50.3% for erythema, compared to −7.7%, −6.1%, and −5.3% for placebo, respectively (all *P* < 0.001). By week 12, reductions reached −91.1%, −90.9%, and −82.3% for xeligekimab versus −18.3%, −20.5%, and −14.9% for placebo (all *P* < 0.001). Following crossover at week 12, placebo-switched patients achieved comparable improvements by week 24 (**Figure 1**; **Supplementary Figure 2**).

**Figure 1:**
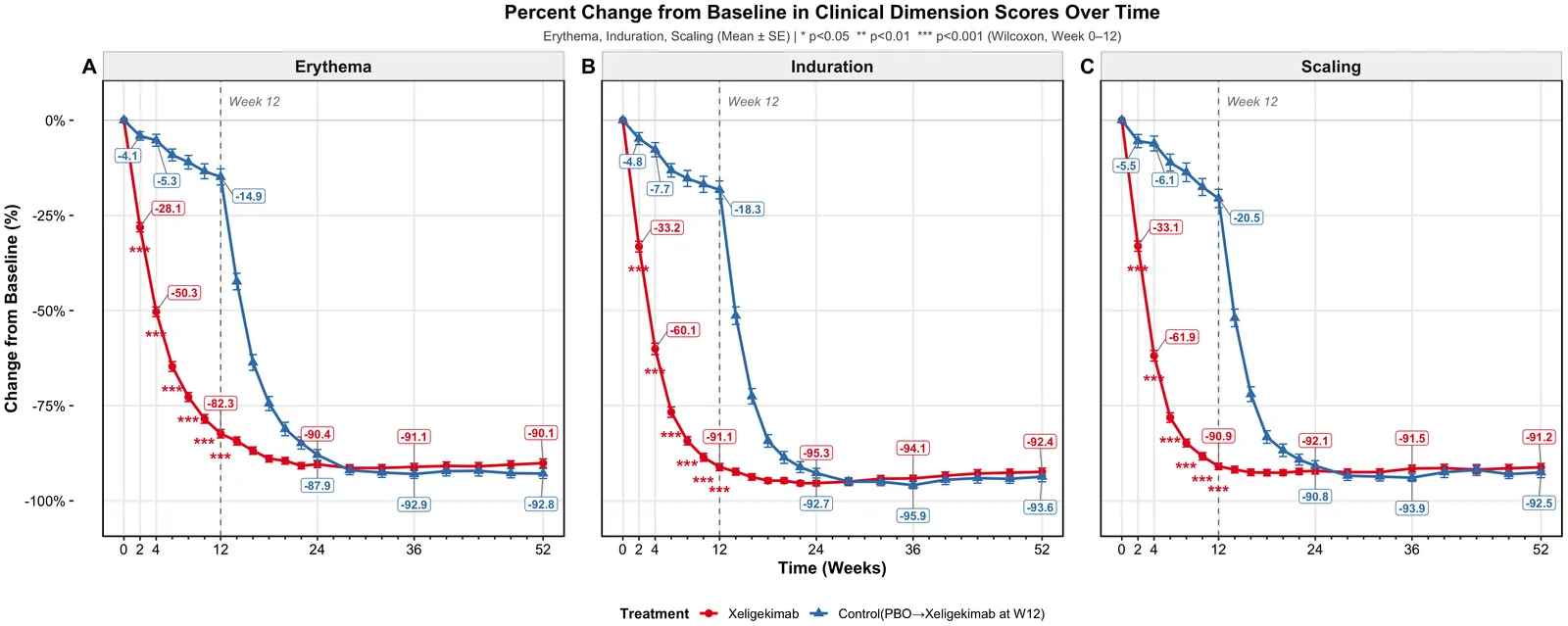
Mean percentage change from baseline in PASI component scores (erythema, induration, and scaling) over time. Mean percentage change (± SE) from baseline in PASI component scores of erythema **(A)**, induration **(B)**, and scaling **(C)** over 52 weeks. Analysis was based on patients with baseline erythema, induration, and scaling scores all >0 (xeligekimab *n* = 281, placebo *n* = 139). Red line: xeligekimab 200 mg Q2W (weeks 0–12) followed by Q4W (weeks 12–52). Blue line: Placebo (weeks 0–12) followed by xeligekimab 200 mg Q4W (weeks 12–52). The vertical dashed line indicates week 12 (crossover point). Between-group comparisons were performed for weeks 0–12 only using the Wilcoxon rank-sum test; *P*-values were adjusted for multiple comparisons using the Benjamini–Hochberg procedure within the comparison family F2 (*m* = 78, encompassing all PASI total, regional, and component score comparisons). ***P < 0.001 for xeligekimab versus placebo. Data points are labeled with mean percentage values. PASI, Psoriasis Area and Severity Index.

Regional analysis is shown in **Figure 2**. Head lesions responded most rapidly, with PASI 90 and PASI 100 rates of 9.5% and 7.2% at week 2 (*P* < 0.01), reaching 84.2% and 71.4% by week 12. The trunk showed similarly robust responses, with PASI 90 of 82.1% and PASI 100 of 66.4% at week 12. Upper limbs achieved PASI 90 of 78.6% and PASI 100 of 67.0%. Lower limbs achieved PASI 90 of 62.7% and PASI 100 of 37.7% at week 12, with continued improvement during the open-label extension to 81.2% and 73.8% by week 52. This is consistent with continued improvement at this typically treatment-resistant anatomical site. All regional comparisons were significant versus placebo at week 12 (all *P* < 0.01). During the open-label extension, responses continued to improve across all regions. By week 52, PASI 90 rates ranged from 81.2% (lower limbs) to 92.1% (trunk), and PASI 100 rates ranged from 73.8% to 89.4%. Placebo-switched patients achieved comparable regional responses by weeks 24–52.

**Figure 2:**
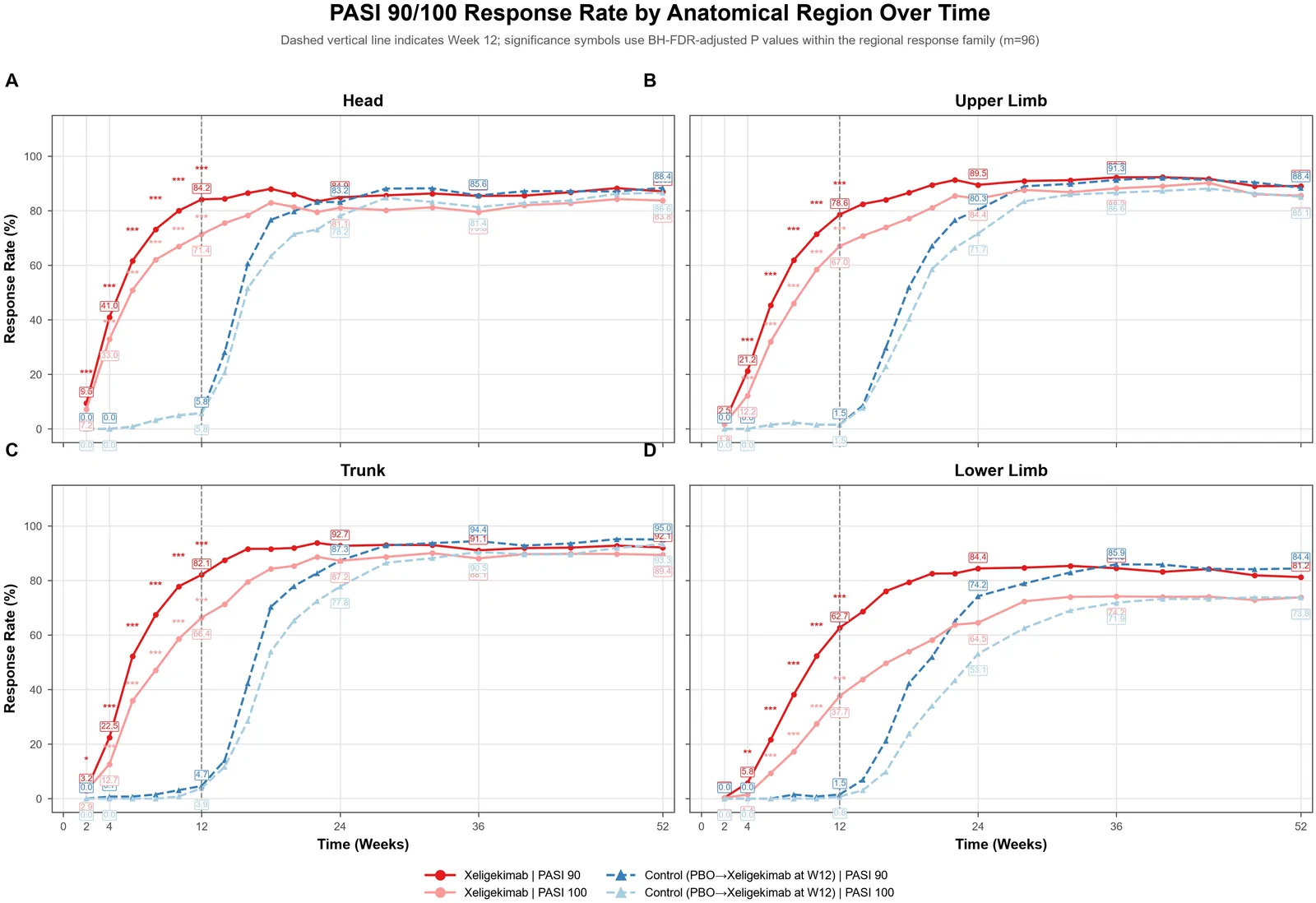
Regional PASI 90 and PASI 100 response rates by anatomical site over time. Proportions of patients achieving regional PASI 90 and PASI 100 in the **(A)** head, **(B)** upper limbs, **(C)** trunk, and **(D)** lower limbs from baseline through week 52. Analysis was based on patients with baseline erythema, induration, and scaling scores >0 at the respective anatomical sites. Eligible sample sizes (xeligekimab/control) were as follows: head, *n* = 264/128; upper limbs, *n* = 281/138; trunk, *n* = 279/137; lower limbs, *n* = 281/139. Solid lines with circles denote xeligekimab 200 mg every 2 weeks during weeks 0–12 followed by 200 mg every 4 weeks during weeks 12–52 (dark red, PASI 90; light red, PASI 100). Dashed lines with triangles denote the control group, which received placebo during weeks 0–12 and crossed over to xeligekimab 200 mg every 4 weeks at week 12 (dark blue, PASI 90; light blue, PASI 100). The vertical dashed line indicates week 12, the point of crossover from placebo to active treatment. Between-group comparisons were performed for weeks 0–12 only using Fisher’s exact test; *P*-values were adjusted for multiple comparisons using the Benjamini–Hochberg procedure within the comparison family F3 (*m* = 96). *P < 0.05, **P < 0.01, and ***P < 0.001 for xeligekimab versus control. Data points represent observed response rates. PASI, Psoriasis Area and Severity Index.

### 3.3 Treatment target achievement

Ideal target achievement rates increased from 1.4% at week 2 to 21.6% at week 4 and 86.6% at week 12, compared to 0%, 0%, and 4.6% for placebo, respectively (all *P* < 0.001 from week 4 onward; **Figure 3A**). Acceptable target rates followed a similar trajectory, reaching 6.4%, 37.8%, and 94.9% at weeks 2, 4, and 12, versus 0.7%, 0.7%, and 9.9% for placebo [*P* < 0.05 at week 2 (Benjamini–Hochberg-adjusted *P* = 0.0109); *P* < 0.001 from week 4 onward; **Figure 3B**]. During the open-label extension, ideal and acceptable target rates continued to improve, peaking at 95.7% and 98.9% at week 24 and remaining at 91.0% and 98.0% through week 52, respectively (**Figures 3A, B**). Placebo-switched patients showed improvement after crossover, achieving ideal target rates of 89.8% and acceptable target rates of 96.9% by week 24, and comparable responses by weeks 36–52.

**Figure 3:**
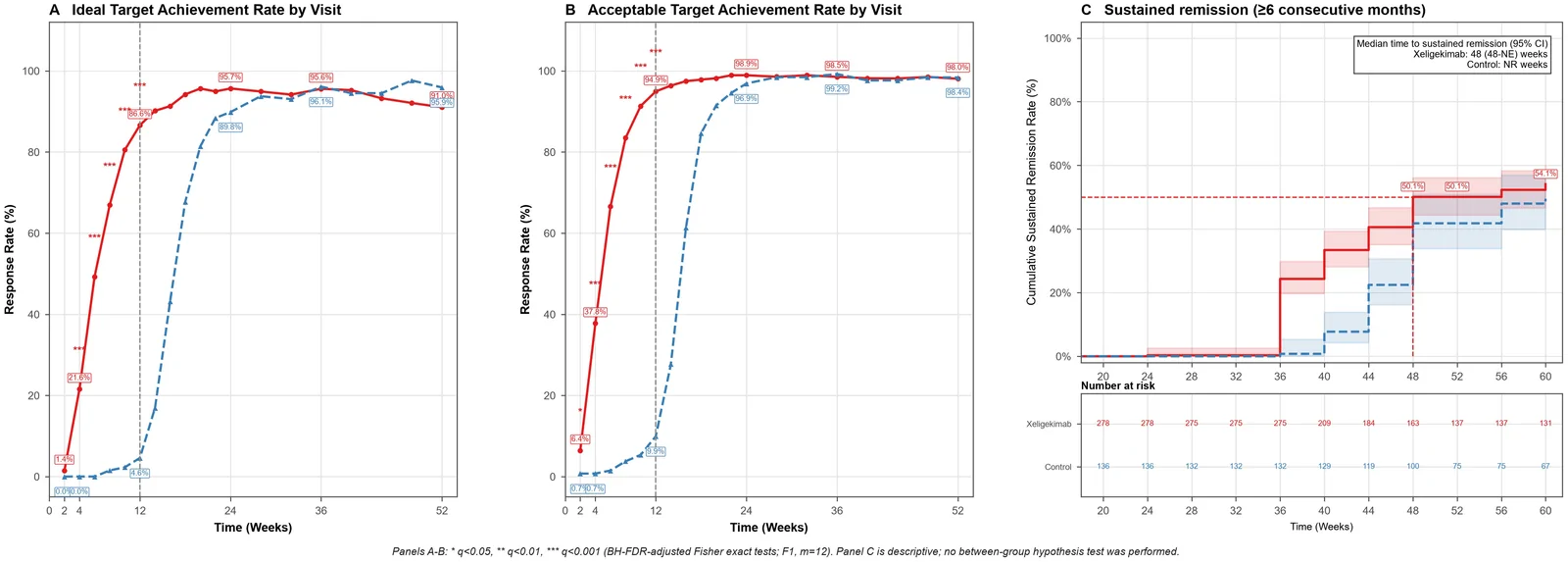
Treatment target achievement and sustained remission rates over time. **(A)** Ideal target and **(B)** acceptable target achievement rates by visit. Solid red lines, xeligekimab (*n* = 281); dashed blue lines, control (placebo weeks 0–12, crossed over to xeligekimab at week 12; *n* = 139). Vertical dashed lines indicate weeks 4 and 12. Between-group comparisons were restricted to weeks 0–12. *P < 0.05, **P < 0.01, and ***P < 0.001 for xeligekimab versus control. **(C)** Kaplan–Meier estimates of cumulative sustained remission. Shaded bands, 95% CI; horizontal dashed line, 50% threshold; Benjamini–Hochberg-adjusted log-rank *P* = 0.040. Numbers at risk are shown below the plot. BSA, body surface area; PASI, Psoriasis Area and Severity Index; PGA, Physician’s Global Assessment.

For sustained remission, the earliest achievement was observed at week 36 in the xeligekimab group (23.7%) (**Figure 3C**). In time-to-event analysis, the median time to sustained remission was 48 weeks (95% CI, 48–not estimable) for xeligekimab-treated patients. By week 52 and week 60, sustained remission rates reached 50.1% and 54.1%, respectively.

### 3.4 Improvement in patient-reported outcomes

Patient-reported outcomes are shown in **Figure 4**. Physical symptom relief was particularly notable, with 91.0% of xeligekimab-treated patients reporting low impact (DLQI Q1 ≤ 1) at week 4 versus 48.0% with placebo (*P* < 0.001) and 82.0% achieving pruritus control (NRS score ≤3) versus 29.0% (*P* < 0.001; **Figure 4A**). By week 12, low-impact rates for xeligekimab exceeded 90% for physical symptoms (97.0% for Q1, 92.0% for pruritus), 98.0% for psychological burden (Q2), and ranged from 97.1% to 98.2% across daily activities dimensions, all significantly higher than placebo (47%–82%, all *P* < 0.001; **Figures 4A–C**). Social functioning showed similarly high rates (Q5: 98.0%, Q8: 98.0%), as did work/study interference (Q7: 97.0%) and treatment burden (Q10: 99.0%), all significantly higher than placebo (71%–77%, all *P* < 0.001; **Figures 4D–F**). Following crossover, placebo-switched patients achieved comparable low-impact rates by week 24 across all dimensions, with both groups maintaining stable rates above 94% through week 52 (all *P* > 0.05).

**Figure 4:**
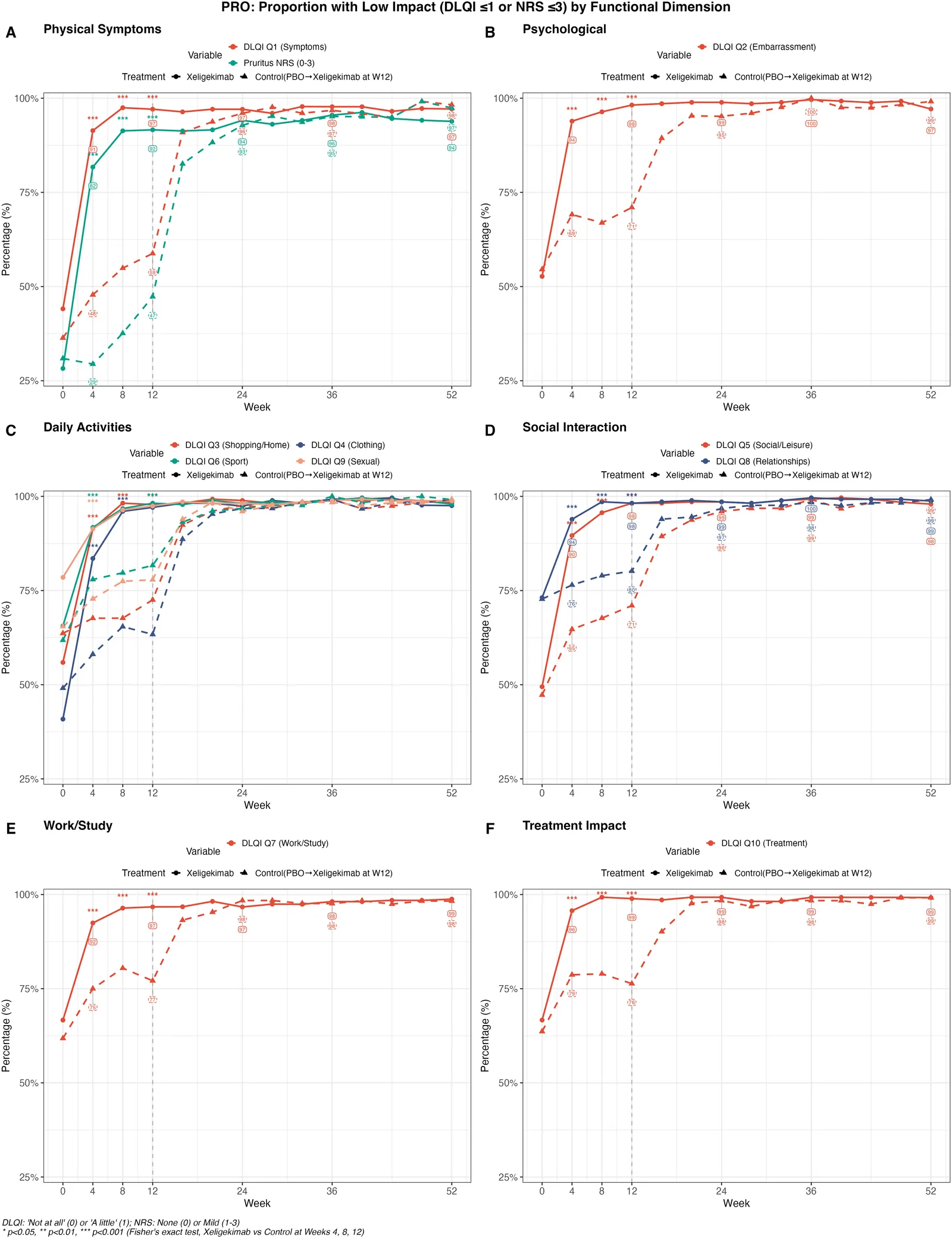
Proportion of patients with low impact across PRO functional dimensions over time. Proportions of patients with low impact across six PRO functional dimensions from baseline through week 52. Low impact: DLQI item score ≤1 or pruritus NRS score ≤3. Panels show individual DLQI items: **(A)** Q1 and pruritus NRS; **(B)** Q2; **(C)** Q3/Q4/Q6/Q9; **(D)** Q5/Q8; **(E)** Q7; **(F)** Q10. Solid lines, xeligekimab (*n* = 281); dashed lines, control (placebo weeks 0–12, crossed over to xeligekimab at week 12; *n* = 139). Vertical dashed line, week 12 (crossover). Between-group comparisons at weeks 4, 8, and 12 only. *P < 0.05, **P < 0.01, and ***P < 0.001 for xeligekimab versus control. DLQI, Dermatology Life Quality Index; NRS, Numerical Rating Scale.

## 4 Discussion

This *post hoc* analysis reveals three key findings that characterize xeligekimab’s multidimensional efficacy profile. Regional response analysis showed differential response kinetics across anatomical sites, with week 52 PASI 90 rates ranging from 81.2% to 92.1% across regions, suggesting broad regional disease control regardless of initial site-specific characteristics. Cumulative treatment target achievement was high, with 86.6% reaching ideal targets and 94.9% reaching acceptable targets by week 12. All six DLQI domains showed comprehensive improvement, with >90% achieving low-impact status by week 12.

The differential regional kinetics observed align with established evidence that anatomical site influences biologic outcomes. Lower extremities represent the most treatment-resistant sites in psoriasis, as demonstrated by Augustin et al. in a topographic analysis of over 2,000 patients (13). Real-world data similarly report persistent lower-limb disease across multiple biologic classes (14). This heterogeneity reflects increased epidermal thickness, reduced vascular density, and mechanical stress in weight-bearing areas (15).

Critically, despite early differences, all regions in our study achieved PASI 90 rates exceeding 62% by week 12 and 81% by week 52. This “catch-up” phenomenon may reflect ongoing *IL-17A* suppression at these treatment-resistant sites. Notably, the lower limb PASI 90 rate of 62.7% at week 12 observed with xeligekimab is within the range reported for other *IL-17* inhibitors at this anatomical site. Regional analyses from the ERASURE/FIXTURE trials of secukinumab reported lower-limb PASI 90 rates of approximately 50%–55% at week 12 (16). Ixekizumab data from UNCOVER showed similar patterns, with lower extremity responses lagging by 10–15 percentage points (17). In a real-world retrospective study, Cheng et al. reported that lower-limb PASI 75 response rates at week 12 were 58.3% for secukinumab and 50.0% for ustekinumab (14). A recent network meta-analysis by Sbidian et al. confirmed that while all *IL-17* inhibitors demonstrate relatively lower-limb resistance, the magnitude of this site-specific gap varies considerably across agents (18). The lower-limb clearance rates achieved with xeligekimab, including the sustained improvement to 81.2% PASI 90 by week 52, are consistent with durable *IL-17A* neutralization in this treatment-resistant region. Additionally, Tada et al. (19) reported in a Japanese real-world cohort that lower-limb PASI improvement lagged other sites by 8–12 weeks across multiple biologics, with week 52 lower-limb PASI 90 rates of 65%–75% for secukinumab and ixekizumab, broadly consistent with the long-term lower-limb efficacy observed with xeligekimab (81.2% at week 52). These cross-trial observations are descriptive and should be interpreted with caution given differences in study populations, designs, and assessment schedules. Clinically, physicians should counsel patients that differential regional responses represent normal trajectories rather than therapeutic failure. Premature treatment switching based on incomplete lower-limb clearance at early time points may deprive patients of eventual full response (20).

Contemporary psoriasis management has shifted toward treat-to-target strategies. The 2023 Chinese expert consensus defined ideal targets as PASI 90 or absolute PASI ≤3 and acceptable targets as PASI 75 or absolute PASI ≤5 (11). National Psoriasis Foundation guidelines similarly advocate goals with regular monitoring (21). Our study operationalized these principles through prespecified targets in this *post hoc* analysis. In translating these targets into practice, appropriate patient selection remains essential, including screening for active infection, latent tuberculosis, and inflammatory bowel disease before initiating *IL-17A* blockade; the safety profile reported in the parent trial should therefore be weighed alongside the efficacy findings presented here (9).

By week 12, ideal target achievement reached 86.6%, increasing to 91.0% by week 52. For context, bimekizumab achieved PASI 90 rates of 85%–90% in BE VIVID/BE READY trials (7, 20), and ixekizumab reached approximately 73% PASI 90 in UNCOVER-3 (22). The high clearance rates with xeligekimab may reflect potent and sustained *IL-17A* neutralization. Sustained remission represents the ultimate therapeutic goal; sustained skin clearance achieved through biologic therapy has been associated with reduced incidence of psoriatic arthritis (23) and lower risk of major adverse cardiovascular events (24) in patients with psoriasis. In our study, median time to sustained remission was 48 weeks, with 54.1% achieving this endpoint by week 60. Considering our stringent definition of sustained remission (continuous complete clearance with BSA = 0% or PGA = 0 for ≥6 consecutive months), these results indicate a durable response. For secukinumab, the pivotal ERASURE and FIXTURE trials reported week 52 PASI 90 responses of 65% and 63%, respectively (16), while the phase 3 CLEAR study demonstrated week 52 PASI 90 and PASI 100 rates of 76.2% and 45.9% (25). Long-term data from the SCULPTURE extension trial further showed that 68.5% of secukinumab responders maintained PASI 90 at year 1 (26). Similarly, for ixekizumab, the UNCOVER-1 and UNCOVER-2 trials reported that among initial responders, 73.3% and 55.3% maintained PASI 90 and PASI 100 at week 60, respectively (17). The IXORA-S trial comparing ixekizumab to ustekinumab showed week 52 PASI 90 and PASI 100 rates of 76.5% and 52.5% (27). Furthermore, 5-year longitudinal data from the UNCOVER trials confirmed the long-term durability of ixekizumab, with sustained complete or near-complete clearance in the majority of adherent patients (28). Given that single-time point PASI 100 (complete clearance) rates for these established *IL-17A* agents typically range between 45% and 55% at 1 year, the 54.1% rate of continuous complete remission (≥6 months) observed with xeligekimab is broadly consistent with these established long-term benchmarks. These comparisons are descriptive and cross-trial in nature and should be interpreted with caution given differences in study populations, definitions, and designs.

Clinical severity measures do not fully capture patient burden. Kimball et al. demonstrated persistent DLQI impairment despite PASI improvement in 30%–40% of patients (29). Strober et al. similarly reported discordance between physician-assessed and patient-perceived disease control (30). This disconnect necessitates domain-specific PRO analysis. All six DLQI domains achieved low-impact rates exceeding 90% by week 12 in our study, which were maintained through week 52. In comparison, the secukinumab CLEAR trial reported DLQI 0/1 rates of 60%–65% at week 16 (25). Brodalumab AMAGINE trials showed DLQI 0/1 rates of approximately 70% at week 12 (31). The higher rates with xeligekimab may reflect comprehensive symptom control beyond visible lesions. Pruritus improvement was particularly rapid, with 82% achieving an NRS score ≤3 by week 4. Ixekizumab UNCOVER data showed approximately 60%–70% achieving significant itch reduction at week 4 (32). This rapid antipruritic effect likely reflects *IL-17A*’s dual role in inflammatory cascades and neurogenic itch. Hawkes et al. demonstrated that *IL-17A* directly activates sensory neurons independent of inflammation (33). Yosipovitch et al. further characterized neuroimmune crosstalk in chronic itch, showing that *IL-17A* upregulates neuropeptide expression in dorsal root ganglia (34). Work productivity (DLQI Q7) and social functioning (Q5/Q8) improvements carry socioeconomic significance. Korman et al. quantified annual productivity losses of 11,000–15,000 per patient with uncontrolled psoriasis (35). Restoration of these domains represents substantial indirect cost savings beyond direct medical expenditures.

Several limitations warrant acknowledgment. First, the *post hoc* design introduces the potential for multiple-comparison bias. Although we applied Benjamini–Hochberg correction within predefined comparison families and observed consistent effect directions across endpoints, residual false-positive risks cannot be excluded. Second, the single-country Chinese population limits generalizability to other ethnic groups, given genetic and environmental heterogeneity in psoriasis phenotypes. Third, the 60-week follow-up provides insufficient data for ultra-long-term efficacy assessment, particularly regarding sustained remission durability beyond 1 year. Fourth, this analysis focused on efficacy and did not evaluate safety, contraindications, or patient-selection criteria, which are essential for treatment decisions.

## 5 Conclusion

This *post hoc* analysis indicates that xeligekimab treatment was associated with rapid, deep, and sustained multidimensional improvements in moderate-to-severe plaque psoriasis. All anatomical regions reached high clearance rates despite early site-specific kinetic differences. Comprehensive improvement across all DLQI domains, particularly early pruritus relief, suggested benefits beyond skin clearance. These findings support further evaluation of xeligekimab as a treatment option, addressing multiple dimensions of psoriasis burden. Prospective studies with biomarker-guided strategies and diverse populations are needed to optimize individualized treatment.

## Data Availability

The datasets generated and analysed during the current study are not publicly available because they are derived from a clinical trial, but are available from the corresponding author upon reasonable request.
