# Supplementary material for: Multidimensional efficacy characterization of xeligekimab in moderate-to-severe plaque psoriasis: regional responses, treatment targets, and patient-reported outcomes from a randomized controlled trial
Source: Front Immunol. 2026 Sep 18;17:1917350. doi: 10.3389/fimmu.2026.1917350 (PMC13630728; doi:10.3389/fimmu.2026.1917350)
Supplement: Supplementary file 1 [file DataSheet1.docx]

**Supplemental Materials**

**Supplemental Figure 1. Mean Change from Baseline in BSA (%) and Total PASI Score Over Time**

(A) Body surface area (BSA, %) and (B) total PASI score, shown as mean change from baseline. Solid lines, Xeligekimab (n=281); dashed lines, control (placebo Weeks 0-12, crossed over to Xeligekimab at Week 12; n=139). Vertical dashed line, Week 12 (crossover). Between-group comparisons at Weeks 2–12 only. **P<0.05, **P<0.01, ***P<0.001* for Xeligekimab versus control. BSA, body surface area; PASI, Psoriasis Area and Severity Index; SE, standard error.

**Supplemental Figure 2. Mean PASI Component Scores (Erythema, Induration, and Scaling) Over Time**

Absolute scores (mean) for (A) erythema, (B) induration, and (C) scaling. Analysis included patients with a baseline score >0 for the corresponding component (xeligekimab n=281, placebo n=139). Solid lines, Xeligekimab (n=281); dashed lines, control (placebo Weeks 0-12, crossed over to Xeligekimab at Week 12; n=139). Vertical dashed line, Week 12 (crossover). Between-group comparisons at Weeks 2-12 only. ****P<0.001* for Xeligekimab versus control. PASI, Psoriasis Area and Severity Index; SE, standard error.

.

**Supplemental Figure 3. Regional PASI Scores by Anatomical Site Over Time**

Absolute regional PASI scores (mean) for (A) head, (B) upper limbs, (C) trunk, and (D) lower limbs. Analysis was restricted to patients with baseline erythema, induration, and scaling scores all >0 at the respective site. Analysis sets (xeligekimab/control) including head, n=264/128; upper limbs, n=281/138; trunk, n=279/137; lower limbs, n=281/139. Solid lines, Xeligekimab; dashed lines, control (placebo Weeks 0–12, crossed over to Xeligekimab at Week 12). Vertical dashed line, Week 12 (crossover). Between-group comparisons at Weeks 2–12 only. ****P<0.001* for Xeligekimab versus control. PASI, Psoriasis Area and Severity Index.

**Supplementary Table 1. Complete Results of Multiple Comparison Testing with Benjamini-Hochberg Correction Across 4 Hypothesis Families**

| **Family** | **Endpoint** | **Time point** | **Test** | **Nominal P** | **BH q** | **m** | **Interpretation** |
| --- | --- | --- | --- | --- | --- | --- | --- |
| F1 | Ideal target | Week 2 | Fisher's exact test | 0.3082 | 0.3082 | 12 | Not significant |
| F1 | Ideal target | Week 4 | Fisher's exact test | <0.001 | <0.001 | 12 | Significant |
| F1 | Ideal target | Week 6 | Fisher's exact test | <0.001 | <0.001 | 12 | Significant |
| F1 | Ideal target | Week 8 | Fisher's exact test | <0.001 | <0.001 | 12 | Significant |
| F1 | Ideal target | Week 10 | Fisher's exact test | <0.001 | <0.001 | 12 | Significant |
| F1 | Ideal target | Week 12 | Fisher's exact test | <0.001 | <0.001 | 12 | Significant |
| F1 | Acceptable target | Week 2 | Fisher's exact test | 0.0100 | 0.0109 | 12 | Significant |
| F1 | Acceptable target | Week 4 | Fisher's exact test | <0.001 | <0.001 | 12 | Significant |
| F1 | Acceptable target | Week 6 | Fisher's exact test | <0.001 | <0.001 | 12 | Significant |
| F1 | Acceptable target | Week 8 | Fisher's exact test | <0.001 | <0.001 | 12 | Significant |
| F1 | Acceptable target | Week 10 | Fisher's exact test | <0.001 | <0.001 | 12 | Significant |
| F1 | Acceptable target | Week 12 | Fisher's exact test | <0.001 | <0.001 | 12 | Significant |
| F2 | PASI Total | Week 2 | Wilcoxon rank-sum test | <0.001 | <0.001 | 78 | Significant |
| F2 | PASI Total | Week 4 | Wilcoxon rank-sum test | <0.001 | <0.001 | 78 | Significant |
| F2 | PASI Total | Week 6 | Wilcoxon rank-sum test | <0.001 | <0.001 | 78 | Significant |
| F2 | PASI Total | Week 8 | Wilcoxon rank-sum test | <0.001 | <0.001 | 78 | Significant |
| F2 | PASI Total | Week 10 | Wilcoxon rank-sum test | <0.001 | <0.001 | 78 | Significant |
| F2 | PASI Total | Week 12 | Wilcoxon rank-sum test | <0.001 | <0.001 | 78 | Significant |
| F2 | Head Score | Week 2 | Wilcoxon rank-sum test | <0.001 | <0.001 | 78 | Significant |
| F2 | Head Score | Week 4 | Wilcoxon rank-sum test | <0.001 | <0.001 | 78 | Significant |
| F2 | Head Score | Week 6 | Wilcoxon rank-sum test | <0.001 | <0.001 | 78 | Significant |
| F2 | Head Score | Week 8 | Wilcoxon rank-sum test | <0.001 | <0.001 | 78 | Significant |
| F2 | Head Score | Week 10 | Wilcoxon rank-sum test | <0.001 | <0.001 | 78 | Significant |
| F2 | Head Score | Week 12 | Wilcoxon rank-sum test | <0.001 | <0.001 | 78 | Significant |
| F2 | Upper Limb Score | Week 2 | Wilcoxon rank-sum test | <0.001 | <0.001 | 78 | Significant |
| F2 | Upper Limb Score | Week 4 | Wilcoxon rank-sum test | <0.001 | <0.001 | 78 | Significant |
| F2 | Upper Limb Score | Week 6 | Wilcoxon rank-sum test | <0.001 | <0.001 | 78 | Significant |
| F2 | Upper Limb Score | Week 8 | Wilcoxon rank-sum test | <0.001 | <0.001 | 78 | Significant |
| F2 | Upper Limb Score | Week 10 | Wilcoxon rank-sum test | <0.001 | <0.001 | 78 | Significant |
| F2 | Upper Limb Score | Week 12 | Wilcoxon rank-sum test | <0.001 | <0.001 | 78 | Significant |
| F2 | Trunk Score | Week 2 | Wilcoxon rank-sum test | <0.001 | <0.001 | 78 | Significant |
| F2 | Trunk Score | Week 4 | Wilcoxon rank-sum test | <0.001 | <0.001 | 78 | Significant |
| F2 | Trunk Score | Week 6 | Wilcoxon rank-sum test | <0.001 | <0.001 | 78 | Significant |
| F2 | Trunk Score | Week 8 | Wilcoxon rank-sum test | <0.001 | <0.001 | 78 | Significant |
| F2 | Trunk Score | Week 10 | Wilcoxon rank-sum test | <0.001 | <0.001 | 78 | Significant |
| F2 | Trunk Score | Week 12 | Wilcoxon rank-sum test | <0.001 | <0.001 | 78 | Significant |
| F2 | Lower Limb Score | Week 2 | Wilcoxon rank-sum test | <0.001 | <0.001 | 78 | Significant |
| F2 | Lower Limb Score | Week 4 | Wilcoxon rank-sum test | <0.001 | <0.001 | 78 | Significant |
| F2 | Lower Limb Score | Week 6 | Wilcoxon rank-sum test | <0.001 | <0.001 | 78 | Significant |
| F2 | Lower Limb Score | Week 8 | Wilcoxon rank-sum test | <0.001 | <0.001 | 78 | Significant |
| F2 | Lower Limb Score | Week 10 | Wilcoxon rank-sum test | <0.001 | <0.001 | 78 | Significant |
| F2 | Lower Limb Score | Week 12 | Wilcoxon rank-sum test | <0.001 | <0.001 | 78 | Significant |
| F2 | Head Area Score | Week 2 | Wilcoxon rank-sum test | <0.001 | <0.001 | 78 | Significant |
| F2 | Head Area Score | Week 4 | Wilcoxon rank-sum test | <0.001 | <0.001 | 78 | Significant |
| F2 | Head Area Score | Week 6 | Wilcoxon rank-sum test | <0.001 | <0.001 | 78 | Significant |
| F2 | Head Area Score | Week 8 | Wilcoxon rank-sum test | <0.001 | <0.001 | 78 | Significant |
| F2 | Head Area Score | Week 10 | Wilcoxon rank-sum test | <0.001 | <0.001 | 78 | Significant |
| F2 | Head Area Score | Week 12 | Wilcoxon rank-sum test | <0.001 | <0.001 | 78 | Significant |
| F2 | Upper Limb Area Score | Week 2 | Wilcoxon rank-sum test | <0.001 | <0.001 | 78 | Significant |
| F2 | Upper Limb Area Score | Week 4 | Wilcoxon rank-sum test | <0.001 | <0.001 | 78 | Significant |
| F2 | Upper Limb Area Score | Week 6 | Wilcoxon rank-sum test | <0.001 | <0.001 | 78 | Significant |
| F2 | Upper Limb Area Score | Week 8 | Wilcoxon rank-sum test | <0.001 | <0.001 | 78 | Significant |
| F2 | Upper Limb Area Score | Week 10 | Wilcoxon rank-sum test | <0.001 | <0.001 | 78 | Significant |
| F2 | Upper Limb Area Score | Week 12 | Wilcoxon rank-sum test | <0.001 | <0.001 | 78 | Significant |
| F2 | Trunk Area Score | Week 2 | Wilcoxon rank-sum test | <0.001 | <0.001 | 78 | Significant |
| F2 | Trunk Area Score | Week 4 | Wilcoxon rank-sum test | <0.001 | <0.001 | 78 | Significant |
| F2 | Trunk Area Score | Week 6 | Wilcoxon rank-sum test | <0.001 | <0.001 | 78 | Significant |
| F2 | Trunk Area Score | Week 8 | Wilcoxon rank-sum test | <0.001 | <0.001 | 78 | Significant |
| F2 | Trunk Area Score | Week 10 | Wilcoxon rank-sum test | <0.001 | <0.001 | 78 | Significant |
| F2 | Trunk Area Score | Week 12 | Wilcoxon rank-sum test | <0.001 | <0.001 | 78 | Significant |
| F2 | Lower Limb Area Score | Week 2 | Wilcoxon rank-sum test | <0.001 | <0.001 | 78 | Significant |
| F2 | Lower Limb Area Score | Week 4 | Wilcoxon rank-sum test | <0.001 | <0.001 | 78 | Significant |
| F2 | Lower Limb Area Score | Week 6 | Wilcoxon rank-sum test | <0.001 | <0.001 | 78 | Significant |
| F2 | Lower Limb Area Score | Week 8 | Wilcoxon rank-sum test | <0.001 | <0.001 | 78 | Significant |
| F2 | Lower Limb Area Score | Week 10 | Wilcoxon rank-sum test | <0.001 | <0.001 | 78 | Significant |
| F2 | Lower Limb Area Score | Week 12 | Wilcoxon rank-sum test | <0.001 | <0.001 | 78 | Significant |
| F2 | Erythema Total | Week 2 | Wilcoxon rank-sum test | <0.001 | <0.001 | 78 | Significant |
| F2 | Erythema Total | Week 4 | Wilcoxon rank-sum test | <0.001 | <0.001 | 78 | Significant |
| F2 | Erythema Total | Week 6 | Wilcoxon rank-sum test | <0.001 | <0.001 | 78 | Significant |
| F2 | Erythema Total | Week 8 | Wilcoxon rank-sum test | <0.001 | <0.001 | 78 | Significant |
| F2 | Erythema Total | Week 10 | Wilcoxon rank-sum test | <0.001 | <0.001 | 78 | Significant |
| F2 | Erythema Total | Week 12 | Wilcoxon rank-sum test | <0.001 | <0.001 | 78 | Significant |
| F2 | Induration Total | Week 2 | Wilcoxon rank-sum test | <0.001 | <0.001 | 78 | Significant |
| F2 | Induration Total | Week 4 | Wilcoxon rank-sum test | <0.001 | <0.001 | 78 | Significant |
| F2 | Induration Total | Week 6 | Wilcoxon rank-sum test | <0.001 | <0.001 | 78 | Significant |
| F2 | Induration Total | Week 8 | Wilcoxon rank-sum test | <0.001 | <0.001 | 78 | Significant |
| F2 | Induration Total | Week 10 | Wilcoxon rank-sum test | <0.001 | <0.001 | 78 | Significant |
| F2 | Induration Total | Week 12 | Wilcoxon rank-sum test | <0.001 | <0.001 | 78 | Significant |
| F2 | Scaling Total | Week 2 | Wilcoxon rank-sum test | <0.001 | <0.001 | 78 | Significant |
| F2 | Scaling Total | Week 4 | Wilcoxon rank-sum test | <0.001 | <0.001 | 78 | Significant |
| F2 | Scaling Total | Week 6 | Wilcoxon rank-sum test | <0.001 | <0.001 | 78 | Significant |
| F2 | Scaling Total | Week 8 | Wilcoxon rank-sum test | <0.001 | <0.001 | 78 | Significant |
| F2 | Scaling Total | Week 10 | Wilcoxon rank-sum test | <0.001 | <0.001 | 78 | Significant |
| F2 | Scaling Total | Week 12 | Wilcoxon rank-sum test | <0.001 | <0.001 | 78 | Significant |
| F2 | BSA (%) | Week 2 | Wilcoxon rank-sum test | <0.001 | <0.001 | 78 | Significant |
| F2 | BSA (%) | Week 4 | Wilcoxon rank-sum test | <0.001 | <0.001 | 78 | Significant |
| F2 | BSA (%) | Week 6 | Wilcoxon rank-sum test | <0.001 | <0.001 | 78 | Significant |
| F2 | BSA (%) | Week 8 | Wilcoxon rank-sum test | <0.001 | <0.001 | 78 | Significant |
| F2 | BSA (%) | Week 10 | Wilcoxon rank-sum test | <0.001 | <0.001 | 78 | Significant |
| F2 | BSA (%) | Week 12 | Wilcoxon rank-sum test | <0.001 | <0.001 | 78 | Significant |
| F3 | Head PASI 50 | Week 2 | Pearson chi-square test | <0.001 | <0.001 | 96 | Significant |
| F3 | Head PASI 50 | Week 4 | Pearson chi-square test | <0.001 | <0.001 | 96 | Significant |
| F3 | Head PASI 50 | Week 6 | Pearson chi-square test | <0.001 | <0.001 | 96 | Significant |
| F3 | Head PASI 50 | Week 8 | Pearson chi-square test | <0.001 | <0.001 | 96 | Significant |
| F3 | Head PASI 50 | Week 10 | Pearson chi-square test | <0.001 | <0.001 | 96 | Significant |
| F3 | Head PASI 50 | Week 12 | Pearson chi-square test | <0.001 | <0.001 | 96 | Significant |
| F3 | Head PASI 75 | Week 2 | Pearson chi-square test | <0.001 | <0.001 | 96 | Significant |
| F3 | Head PASI 75 | Week 4 | Pearson chi-square test | <0.001 | <0.001 | 96 | Significant |
| F3 | Head PASI 75 | Week 6 | Pearson chi-square test | <0.001 | <0.001 | 96 | Significant |
| F3 | Head PASI 75 | Week 8 | Pearson chi-square test | <0.001 | <0.001 | 96 | Significant |
| F3 | Head PASI 75 | Week 10 | Pearson chi-square test | <0.001 | <0.001 | 96 | Significant |
| F3 | Head PASI 75 | Week 12 | Pearson chi-square test | <0.001 | <0.001 | 96 | Significant |
| F3 | Head PASI 90 | Week 2 | Pearson chi-square test | <0.001 | <0.001 | 96 | Significant |
| F3 | Head PASI 90 | Week 4 | Pearson chi-square test | <0.001 | <0.001 | 96 | Significant |
| F3 | Head PASI 90 | Week 6 | Pearson chi-square test | <0.001 | <0.001 | 96 | Significant |
| F3 | Head PASI 90 | Week 8 | Pearson chi-square test | <0.001 | <0.001 | 96 | Significant |
| F3 | Head PASI 90 | Week 10 | Pearson chi-square test | <0.001 | <0.001 | 96 | Significant |
| F3 | Head PASI 90 | Week 12 | Pearson chi-square test | <0.001 | <0.001 | 96 | Significant |
| F3 | Head PASI 100 | Week 2 | Pearson chi-square test | 0.0021 | 0.0023 | 96 | Significant |
| F3 | Head PASI 100 | Week 4 | Pearson chi-square test | <0.001 | <0.001 | 96 | Significant |
| F3 | Head PASI 100 | Week 6 | Pearson chi-square test | <0.001 | <0.001 | 96 | Significant |
| F3 | Head PASI 100 | Week 8 | Pearson chi-square test | <0.001 | <0.001 | 96 | Significant |
| F3 | Head PASI 100 | Week 10 | Pearson chi-square test | <0.001 | <0.001 | 96 | Significant |
| F3 | Head PASI 100 | Week 12 | Pearson chi-square test | <0.001 | <0.001 | 96 | Significant |
| F3 | Upper Limb PASI 50 | Week 2 | Pearson chi-square test | <0.001 | <0.001 | 96 | Significant |
| F3 | Upper Limb PASI 50 | Week 4 | Pearson chi-square test | <0.001 | <0.001 | 96 | Significant |
| F3 | Upper Limb PASI 50 | Week 6 | Pearson chi-square test | <0.001 | <0.001 | 96 | Significant |
| F3 | Upper Limb PASI 50 | Week 8 | Pearson chi-square test | <0.001 | <0.001 | 96 | Significant |
| F3 | Upper Limb PASI 50 | Week 10 | Pearson chi-square test | <0.001 | <0.001 | 96 | Significant |
| F3 | Upper Limb PASI 50 | Week 12 | Pearson chi-square test | <0.001 | <0.001 | 96 | Significant |
| F3 | Upper Limb PASI 75 | Week 2 | Pearson chi-square test | 0.0026 | 0.0029 | 96 | Significant |
| F3 | Upper Limb PASI 75 | Week 4 | Pearson chi-square test | <0.001 | <0.001 | 96 | Significant |
| F3 | Upper Limb PASI 75 | Week 6 | Pearson chi-square test | <0.001 | <0.001 | 96 | Significant |
| F3 | Upper Limb PASI 75 | Week 8 | Pearson chi-square test | <0.001 | <0.001 | 96 | Significant |
| F3 | Upper Limb PASI 75 | Week 10 | Pearson chi-square test | <0.001 | <0.001 | 96 | Significant |
| F3 | Upper Limb PASI 75 | Week 12 | Pearson chi-square test | <0.001 | <0.001 | 96 | Significant |
| F3 | Upper Limb PASI 90 | Week 2 | Fisher's exact test | 0.1016 | 0.1072 | 96 | Not significant |
| F3 | Upper Limb PASI 90 | Week 4 | Pearson chi-square test | <0.001 | <0.001 | 96 | Significant |
| F3 | Upper Limb PASI 90 | Week 6 | Pearson chi-square test | <0.001 | <0.001 | 96 | Significant |
| F3 | Upper Limb PASI 90 | Week 8 | Pearson chi-square test | <0.001 | <0.001 | 96 | Significant |
| F3 | Upper Limb PASI 90 | Week 10 | Pearson chi-square test | <0.001 | <0.001 | 96 | Significant |
| F3 | Upper Limb PASI 90 | Week 12 | Pearson chi-square test | <0.001 | <0.001 | 96 | Significant |
| F3 | Upper Limb PASI 100 | Week 2 | Fisher's exact test | 0.1787 | 0.1864 | 96 | Not significant |
| F3 | Upper Limb PASI 100 | Week 4 | Pearson chi-square test | <0.001 | <0.001 | 96 | Significant |
| F3 | Upper Limb PASI 100 | Week 6 | Pearson chi-square test | <0.001 | <0.001 | 96 | Significant |
| F3 | Upper Limb PASI 100 | Week 8 | Pearson chi-square test | <0.001 | <0.001 | 96 | Significant |
| F3 | Upper Limb PASI 100 | Week 10 | Pearson chi-square test | <0.001 | <0.001 | 96 | Significant |
| F3 | Upper Limb PASI 100 | Week 12 | Pearson chi-square test | <0.001 | <0.001 | 96 | Significant |
| F3 | Trunk PASI 50 | Week 2 | Pearson chi-square test | <0.001 | <0.001 | 96 | Significant |
| F3 | Trunk PASI 50 | Week 4 | Pearson chi-square test | <0.001 | <0.001 | 96 | Significant |
| F3 | Trunk PASI 50 | Week 6 | Pearson chi-square test | <0.001 | <0.001 | 96 | Significant |
| F3 | Trunk PASI 50 | Week 8 | Pearson chi-square test | <0.001 | <0.001 | 96 | Significant |
| F3 | Trunk PASI 50 | Week 10 | Pearson chi-square test | <0.001 | <0.001 | 96 | Significant |
| F3 | Trunk PASI 50 | Week 12 | Pearson chi-square test | <0.001 | <0.001 | 96 | Significant |
| F3 | Trunk PASI 75 | Week 2 | Pearson chi-square test | <0.001 | <0.001 | 96 | Significant |
| F3 | Trunk PASI 75 | Week 4 | Pearson chi-square test | <0.001 | <0.001 | 96 | Significant |
| F3 | Trunk PASI 75 | Week 6 | Pearson chi-square test | <0.001 | <0.001 | 96 | Significant |
| F3 | Trunk PASI 75 | Week 8 | Pearson chi-square test | <0.001 | <0.001 | 96 | Significant |
| F3 | Trunk PASI 75 | Week 10 | Pearson chi-square test | <0.001 | <0.001 | 96 | Significant |
| F3 | Trunk PASI 75 | Week 12 | Pearson chi-square test | <0.001 | <0.001 | 96 | Significant |
| F3 | Trunk PASI 90 | Week 2 | Fisher's exact test | 0.0343 | 0.0370 | 96 | Significant |
| F3 | Trunk PASI 90 | Week 4 | Pearson chi-square test | <0.001 | <0.001 | 96 | Significant |
| F3 | Trunk PASI 90 | Week 6 | Pearson chi-square test | <0.001 | <0.001 | 96 | Significant |
| F3 | Trunk PASI 90 | Week 8 | Pearson chi-square test | <0.001 | <0.001 | 96 | Significant |
| F3 | Trunk PASI 90 | Week 10 | Pearson chi-square test | <0.001 | <0.001 | 96 | Significant |
| F3 | Trunk PASI 90 | Week 12 | Pearson chi-square test | <0.001 | <0.001 | 96 | Significant |
| F3 | Trunk PASI 100 | Week 2 | Fisher's exact test | 0.0579 | 0.0618 | 96 | Not significant |
| F3 | Trunk PASI 100 | Week 4 | Pearson chi-square test | <0.001 | <0.001 | 96 | Significant |
| F3 | Trunk PASI 100 | Week 6 | Pearson chi-square test | <0.001 | <0.001 | 96 | Significant |
| F3 | Trunk PASI 100 | Week 8 | Pearson chi-square test | <0.001 | <0.001 | 96 | Significant |
| F3 | Trunk PASI 100 | Week 10 | Pearson chi-square test | <0.001 | <0.001 | 96 | Significant |
| F3 | Trunk PASI 100 | Week 12 | Pearson chi-square test | <0.001 | <0.001 | 96 | Significant |
| F3 | Lower Limb PASI 50 | Week 2 | Pearson chi-square test | <0.001 | <0.001 | 96 | Significant |
| F3 | Lower Limb PASI 50 | Week 4 | Pearson chi-square test | <0.001 | <0.001 | 96 | Significant |
| F3 | Lower Limb PASI 50 | Week 6 | Pearson chi-square test | <0.001 | <0.001 | 96 | Significant |
| F3 | Lower Limb PASI 50 | Week 8 | Pearson chi-square test | <0.001 | <0.001 | 96 | Significant |
| F3 | Lower Limb PASI 50 | Week 10 | Pearson chi-square test | <0.001 | <0.001 | 96 | Significant |
| F3 | Lower Limb PASI 50 | Week 12 | Pearson chi-square test | <0.001 | <0.001 | 96 | Significant |
| F3 | Lower Limb PASI 75 | Week 2 | Fisher's exact test | 0.1837 | 0.1896 | 96 | Not significant |
| F3 | Lower Limb PASI 75 | Week 4 | Pearson chi-square test | <0.001 | <0.001 | 96 | Significant |
| F3 | Lower Limb PASI 75 | Week 6 | Pearson chi-square test | <0.001 | <0.001 | 96 | Significant |
| F3 | Lower Limb PASI 75 | Week 8 | Pearson chi-square test | <0.001 | <0.001 | 96 | Significant |
| F3 | Lower Limb PASI 75 | Week 10 | Pearson chi-square test | <0.001 | <0.001 | 96 | Significant |
| F3 | Lower Limb PASI 75 | Week 12 | Pearson chi-square test | <0.001 | <0.001 | 96 | Significant |
| F3 | Lower Limb PASI 90 | Week 2 | Fisher's exact test | 1.0000 | 1.0000 | 96 | Not significant |
| F3 | Lower Limb PASI 90 | Week 4 | Pearson chi-square test | 0.0043 | 0.0047 | 96 | Significant |
| F3 | Lower Limb PASI 90 | Week 6 | Pearson chi-square test | <0.001 | <0.001 | 96 | Significant |
| F3 | Lower Limb PASI 90 | Week 8 | Pearson chi-square test | <0.001 | <0.001 | 96 | Significant |
| F3 | Lower Limb PASI 90 | Week 10 | Pearson chi-square test | <0.001 | <0.001 | 96 | Significant |
| F3 | Lower Limb PASI 90 | Week 12 | Pearson chi-square test | <0.001 | <0.001 | 96 | Significant |
| F3 | Lower Limb PASI 100 | Week 2 | Fisher's exact test | 1.0000 | 1.0000 | 96 | Not significant |
| F3 | Lower Limb PASI 100 | Week 4 | Fisher's exact test | 0.3077 | 0.3142 | 96 | Not significant |
| F3 | Lower Limb PASI 100 | Week 6 | Pearson chi-square test | <0.001 | <0.001 | 96 | Significant |
| F3 | Lower Limb PASI 100 | Week 8 | Pearson chi-square test | <0.001 | <0.001 | 96 | Significant |
| F3 | Lower Limb PASI 100 | Week 10 | Pearson chi-square test | <0.001 | <0.001 | 96 | Significant |
| F3 | Lower Limb PASI 100 | Week 12 | Pearson chi-square test | <0.001 | <0.001 | 96 | Significant |
| F4 | Pruritus NRS (0-10): ordinal distribution | Week 4 | Wilcoxon rank-sum test | <0.001 | <0.001 | 63 | Significant |
| F4 | Pruritus NRS (0-10): ordinal distribution | Week 8 | Wilcoxon rank-sum test | <0.001 | <0.001 | 63 | Significant |
| F4 | Pruritus NRS (0-10): ordinal distribution | Week 12 | Wilcoxon rank-sum test | <0.001 | <0.001 | 63 | Significant |
| F4 | DLQI Q1 (Symptoms): ordinal distribution | Week 4 | Wilcoxon rank-sum test | <0.001 | <0.001 | 63 | Significant |
| F4 | DLQI Q1 (Symptoms): ordinal distribution | Week 8 | Wilcoxon rank-sum test | <0.001 | <0.001 | 63 | Significant |
| F4 | DLQI Q1 (Symptoms): ordinal distribution | Week 12 | Wilcoxon rank-sum test | <0.001 | <0.001 | 63 | Significant |
| F4 | DLQI Q2 (Embarrassment): ordinal distribution | Week 4 | Wilcoxon rank-sum test | <0.001 | <0.001 | 63 | Significant |
| F4 | DLQI Q2 (Embarrassment): ordinal distribution | Week 8 | Wilcoxon rank-sum test | <0.001 | <0.001 | 63 | Significant |
| F4 | DLQI Q2 (Embarrassment): ordinal distribution | Week 12 | Wilcoxon rank-sum test | <0.001 | <0.001 | 63 | Significant |
| F4 | DLQI Q3 (Shopping/Home): ordinal distribution | Week 4 | Wilcoxon rank-sum test | <0.001 | <0.001 | 63 | Significant |
| F4 | DLQI Q3 (Shopping/Home): ordinal distribution | Week 8 | Wilcoxon rank-sum test | <0.001 | <0.001 | 63 | Significant |
| F4 | DLQI Q3 (Shopping/Home): ordinal distribution | Week 12 | Wilcoxon rank-sum test | <0.001 | <0.001 | 63 | Significant |
| F4 | DLQI Q4 (Clothing): ordinal distribution | Week 4 | Wilcoxon rank-sum test | <0.001 | <0.001 | 63 | Significant |
| F4 | DLQI Q4 (Clothing): ordinal distribution | Week 8 | Wilcoxon rank-sum test | <0.001 | <0.001 | 63 | Significant |
| F4 | DLQI Q4 (Clothing): ordinal distribution | Week 12 | Wilcoxon rank-sum test | <0.001 | <0.001 | 63 | Significant |
| F4 | DLQI Q5 (Social/Leisure): ordinal distribution | Week 4 | Wilcoxon rank-sum test | <0.001 | <0.001 | 63 | Significant |
| F4 | DLQI Q5 (Social/Leisure): ordinal distribution | Week 8 | Wilcoxon rank-sum test | <0.001 | <0.001 | 63 | Significant |
| F4 | DLQI Q5 (Social/Leisure): ordinal distribution | Week 12 | Wilcoxon rank-sum test | <0.001 | <0.001 | 63 | Significant |
| F4 | DLQI Q6 (Sport): ordinal distribution | Week 4 | Wilcoxon rank-sum test | <0.001 | <0.001 | 63 | Significant |
| F4 | DLQI Q6 (Sport): ordinal distribution | Week 8 | Wilcoxon rank-sum test | <0.001 | <0.001 | 63 | Significant |
| F4 | DLQI Q6 (Sport): ordinal distribution | Week 12 | Wilcoxon rank-sum test | <0.001 | <0.001 | 63 | Significant |
| F4 | DLQI Q8 (Relationships): ordinal distribution | Week 4 | Wilcoxon rank-sum test | <0.001 | <0.001 | 63 | Significant |
| F4 | DLQI Q8 (Relationships): ordinal distribution | Week 8 | Wilcoxon rank-sum test | <0.001 | <0.001 | 63 | Significant |
| F4 | DLQI Q8 (Relationships): ordinal distribution | Week 12 | Wilcoxon rank-sum test | <0.001 | <0.001 | 63 | Significant |
| F4 | DLQI Q9 (Sexual): ordinal distribution | Week 4 | Wilcoxon rank-sum test | <0.001 | <0.001 | 63 | Significant |
| F4 | DLQI Q9 (Sexual): ordinal distribution | Week 8 | Wilcoxon rank-sum test | <0.001 | <0.001 | 63 | Significant |
| F4 | DLQI Q9 (Sexual): ordinal distribution | Week 12 | Wilcoxon rank-sum test | <0.001 | <0.001 | 63 | Significant |
| F4 | DLQI Q10 (Treatment): ordinal distribution | Week 4 | Wilcoxon rank-sum test | <0.001 | <0.001 | 63 | Significant |
| F4 | DLQI Q10 (Treatment): ordinal distribution | Week 8 | Wilcoxon rank-sum test | <0.001 | <0.001 | 63 | Significant |
| F4 | DLQI Q10 (Treatment): ordinal distribution | Week 12 | Wilcoxon rank-sum test | <0.001 | <0.001 | 63 | Significant |
| F4 | DLQI Q1 (Symptoms): low impact | Week 4 | Fisher's exact test | <0.001 | <0.001 | 63 | Significant |
| F4 | DLQI Q1 (Symptoms): low impact | Week 8 | Fisher's exact test | <0.001 | <0.001 | 63 | Significant |
| F4 | DLQI Q1 (Symptoms): low impact | Week 12 | Fisher's exact test | <0.001 | <0.001 | 63 | Significant |
| F4 | Pruritus NRS (0-3): low impact | Week 4 | Fisher's exact test | <0.001 | <0.001 | 63 | Significant |
| F4 | Pruritus NRS (0-3): low impact | Week 8 | Fisher's exact test | <0.001 | <0.001 | 63 | Significant |
| F4 | Pruritus NRS (0-3): low impact | Week 12 | Fisher's exact test | <0.001 | <0.001 | 63 | Significant |
| F4 | DLQI Q2 (Embarrassment): low impact | Week 4 | Fisher's exact test | <0.001 | <0.001 | 63 | Significant |
| F4 | DLQI Q2 (Embarrassment): low impact | Week 8 | Fisher's exact test | <0.001 | <0.001 | 63 | Significant |
| F4 | DLQI Q2 (Embarrassment): low impact | Week 12 | Fisher's exact test | <0.001 | <0.001 | 63 | Significant |
| F4 | DLQI Q3 (Shopping/Home): low impact | Week 4 | Fisher's exact test | <0.001 | <0.001 | 63 | Significant |
| F4 | DLQI Q3 (Shopping/Home): low impact | Week 8 | Fisher's exact test | <0.001 | <0.001 | 63 | Significant |
| F4 | DLQI Q3 (Shopping/Home): low impact | Week 12 | Fisher's exact test | <0.001 | <0.001 | 63 | Significant |
| F4 | DLQI Q4 (Clothing): low impact | Week 4 | Fisher's exact test | <0.001 | <0.001 | 63 | Significant |
| F4 | DLQI Q4 (Clothing): low impact | Week 8 | Fisher's exact test | <0.001 | <0.001 | 63 | Significant |
| F4 | DLQI Q4 (Clothing): low impact | Week 12 | Fisher's exact test | <0.001 | <0.001 | 63 | Significant |
| F4 | DLQI Q6 (Sport): low impact | Week 4 | Fisher's exact test | <0.001 | <0.001 | 63 | Significant |
| F4 | DLQI Q6 (Sport): low impact | Week 8 | Fisher's exact test | <0.001 | <0.001 | 63 | Significant |
| F4 | DLQI Q6 (Sport): low impact | Week 12 | Fisher's exact test | <0.001 | <0.001 | 63 | Significant |
| F4 | DLQI Q9 (Sexual): low impact | Week 4 | Fisher's exact test | <0.001 | <0.001 | 63 | Significant |
| F4 | DLQI Q9 (Sexual): low impact | Week 8 | Fisher's exact test | <0.001 | <0.001 | 63 | Significant |
| F4 | DLQI Q9 (Sexual): low impact | Week 12 | Fisher's exact test | <0.001 | <0.001 | 63 | Significant |
| F4 | DLQI Q5 (Social/Leisure): low impact | Week 4 | Fisher's exact test | <0.001 | <0.001 | 63 | Significant |
| F4 | DLQI Q5 (Social/Leisure): low impact | Week 8 | Fisher's exact test | <0.001 | <0.001 | 63 | Significant |
| F4 | DLQI Q5 (Social/Leisure): low impact | Week 12 | Fisher's exact test | <0.001 | <0.001 | 63 | Significant |
| F4 | DLQI Q8 (Relationships): low impact | Week 4 | Fisher's exact test | <0.001 | <0.001 | 63 | Significant |
| F4 | DLQI Q8 (Relationships): low impact | Week 8 | Fisher's exact test | <0.001 | <0.001 | 63 | Significant |
| F4 | DLQI Q8 (Relationships): low impact | Week 12 | Fisher's exact test | <0.001 | <0.001 | 63 | Significant |
| F4 | DLQI Q7 (Work/Study): low impact | Week 4 | Fisher's exact test | <0.001 | <0.001 | 63 | Significant |
| F4 | DLQI Q7 (Work/Study): low impact | Week 8 | Fisher's exact test | <0.001 | <0.001 | 63 | Significant |
| F4 | DLQI Q7 (Work/Study): low impact | Week 12 | Fisher's exact test | <0.001 | <0.001 | 63 | Significant |
| F4 | DLQI Q10 (Treatment): low impact | Week 4 | Fisher's exact test | <0.001 | <0.001 | 63 | Significant |
| F4 | DLQI Q10 (Treatment): low impact | Week 8 | Fisher's exact test | <0.001 | <0.001 | 63 | Significant |
| F4 | DLQI Q10 (Treatment): low impact | Week 12 | Fisher's exact test | <0.001 | <0.001 | 63 | Significant |
